# Non-steroidal anti-inflammatory drug – induced transient reactive papulotranslucent acrokeratoderma

**Published:** 2014-03-25

**Authors:** OA Orzan, LG Popa, V Voiculescu, R Manta, C Giurcăneanu

**Affiliations:** * "Carol Davila" University of Medicine and Pharmacy, Bucharest; ** "Elias" Emergency Hospital, Department of Dermatology

**Keywords:** non-steroidal, papulotranslucent acrokeratoderma, palmar wrinkling

## Abstract

Transient reactive papulotranslucent acrokeratoderma (TRPA) is an unusual skin condition characterized by the rapid and transient development of symmetric, edematous white papules with eccrine duct prominence on the palms after exposure to water. We present the case of a 28-year-old woman diagnosed in our clinic with TRPA induced by the use of a non-steroidal anti-inflammatory drug. The possible pathophysiology and treatment options are discussed.

## Introduction

Transient reactive papulotranslucent acrokeratoderma (TRPA), also known as acquired aquagenic palmoplantar keratoderma, aquagenic keratoderma, aquagenic wrinkling of the palms, and aquagenic syringeal acrokeratoderma [**1**] is an unusual skin condition characterized by the rapid and transient development of symmetric, edematous white papules with eccrine duct prominence on the palms after exposure to water. It is a rare disorder, fewer than 40 cases having been reported in literature. It mainly affects young women, reported patients being aged between 6 and 50 years. 

 The majority of reported cases of TRPA have associated cystic fibrosis, the δF508 CFTR (cystic fibrosis transmembrane conductance regulator) mutation being thought to be a predisposing factor for this disease [**2**]. A cystic fibrosis carrier state, a cutaneous barrier function defect, hyperhidrosis and the intake of cyclooxygenase inhibitors may also be pathogenic factors.

## Case report

We present the case of a 28-year-old woman who was evaluated in our clinic for a several-weeks history of exaggerated palmar wrinkling and swelling after 3-4 minutes exposure of her hands to water. She reported having experienced the same signs and symptoms the previous year for approximately 3 weeks, but the abnormalities healed spontaneously after this interval with diffuse descuamation of the palmar surfaces.

**Fig. 1:**
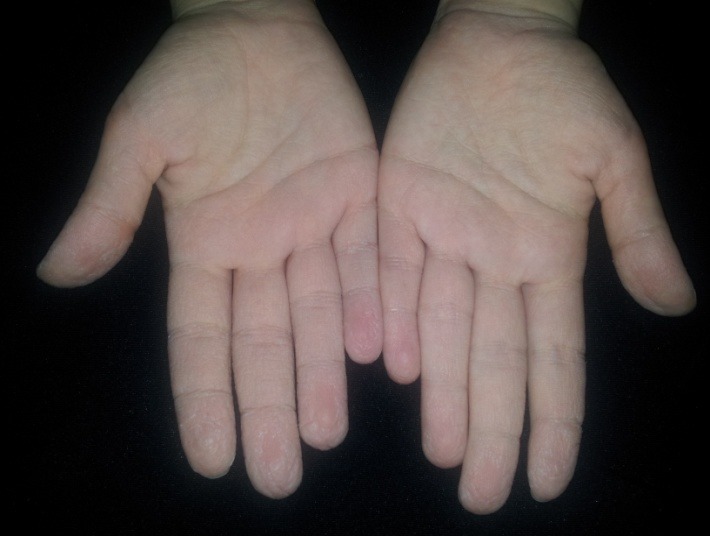
Exaggerated palmar wrinkling

Physical examination revealed normal skin on her palms. The rest of the general physical examination did not reveal pathologic changes. Following brief exposure to water at room temperature (4 minutes), she developed swelling and a whitish discoloration of the palms, followed by the appearance of 1-2 mm white, translucent papules, coalescing into pebbly plaques distributed diffusely on the palmar surfaces, consistent with a positive “hand in bucket" sign. In addition, the eccrine pores were more prominent. The patient complained of local disconfort during the episode. After drying, the signs and symptoms resolved spontaneously within thirty minutes. 

 The patient did not associate palmoplantar hyperhidrosis. She was not aware of any family history of TRPA or cystic fibrosis and tested negative for δF508 CFTR mutation. She refused skin biopsy. 

 The patient denied any concomitant medication except for Ketorolac, a non-steroidal anti-inflammatory drug, which she occasionally administered for low back pain during the previous month. When asked, the patient admitted to have taken Ketorolac for the same condition the previous year and correlated the appearance of the described cutaneous signs with the initiation of the anti-inflammatory treatment.

 Treatment with Ketorolac was stopped. The patient was advised to avoid water exposure as much as possible and to wear gloves. She was recommended 9% aluminum chloride cream and a barrier hand cream that resulted in a slight improvement of the symptoms. One week following the discontinuation of the anti-inflammatory therapy, the patient presented diffuse desquamation of the palms. Afterwards, the aquagenic palmar lesions ceased to appear. 

## Discussion

 Two types of papulotranslucent acrokeratoderma have been described in literature, hereditary and transient reactive. 

 Onwukwe et al. were the first to describe the condition in 1973, as hereditary papulotranslucent acrokeratoderma (HPA), an autosomal dominant disease presenting with persistent asymptomatic bilateral, translucent, yellowish-white papules and plaques located especially on areas of increased pressure and trauma on the palms and soles. Similar cases were later reported by Koster and Nasemann in 1985 and Heymann in 1998 [**3,4**]. An association with atopy and fine-textured scalp hair was observed in these cases. The pathogenesis of HPA is unknown. As only palms and soles are affected, it was hypothesized that physical trauma is an initiating factor [**5**]. Specific treatments for HPA are not usually necessary because the condition is generally asymptomatic [**6**]. 

 In 1996, English and McCollough reported the first cases of transient reactive papulotranslucent acrokeratoderma. They described two sisters with episodic development of white papules and plaques on the palms that became edematous and painful following exposure to water [**6,8,9-12**]. Since then, a few other cases with the aquired form of disease have been described [**7**]. In contrast to the hereditary form, these patients presented with transient lesions that were not limited to pressure areas of the palms and had no history of atopy. 

 The etiopathogenesis of TRPA is still unclear. An association with cystic fibrosis and the carrier state of the cystic fibrosis gene has been suggested [**13**]. The clinical manifestations may be caused by an increased epidermal sodium level, leading to increased water retention within keratinocytes. Abnormal regulation by CFTR of cell-membrane water channels, such as aquaporin 3, defective stratum corneum barrier function, occlusion of the eccrine duct ostia, or weakness of the eccrine duct wall have also been incriminated [**2**]. Moreover, TRPA secondary to the use of non-steroidal anti-inflammatory drugs (aspirin [**14**] and rofecoxib [**15**]) has been described. Cyclooxygenase-2 (COX-2) inhibitors increase sodium reabsorption in the kidney by their effects on renal vasculature. COX-2 is also present in keratinocytes and its inhibition may cause increased sodium reabsorption and, subsequently, increased keratin water-binding capacity [**14**].

 Clinically, TRPA is characterized by the appearance of white or translucent papules that coalesce into pebbly plaques on the palmar surfaces following short exposure to water. Soles are usually unaffected. The changes may be asymptomatic or accompanied by local discomfort, pruritus, a burning sensation or even pain. In most cases, the palmar skin returns to normal within a few minutes after drying. Sometimes, patients also suffer from palmoplantar hyperhidrosis. 

 The diagnosis of TRPA is usually made on the clinical history and physical findings. The most common histologic findings are hyperkeratosis and dilated eccrine ostia [**7,9**].

 TRPA treatment is generally disappointing. Treatment options include providing a water barrier to prevent exposure and aluminum chloride products. Use of barrier creams such as hydrophilic petrolatum, glycerin emollients, 5-20% salicylic acid, 10% urea, and 12% ammonium lactate creams has not been shown to be effective [9,10]. Patients with associated palmar hyperhidrosis benefit from aluminum chloride-containing products [**6**].

## Conclusions

The patient presented demonstrates a new case of TRPA. The clinical manifestations are transient and the reactivity to water is a prominent feature of the aquired form of the disease [**9**]. None of the patient’s relatives present similar symptoms, nor a history of atopy. 

 The most probable cause of TRPA in our patient was the use of non-steroidal anti-inflammatory drugs. This hypotesis is sustained by the temporal correlation between the onset of the cutaneous abnormalities and the initiation of the anti-inflammatory treatment, as well as by the complete clinical remission upon discontinuation of this treatment. 

 Cystic fibrosis or the carrier state of cystic fibrosis gene should be considered in patients with TRPA, and patients with cystic fibrosis should be asked about symptoms of this condition. Patient interview should also focus on the use of non-steroidal anti-inflammatory drugs, as discontinuing this type of medication could lead to clinical improvement. 

 Further observations are needed in order to elucidate the etiopathogenesis of this condition and to develop more efficient treatment modalities.
